# Sevoflurane preconditioning induced endogenous neurogenesis against ischemic brain injury by promoting microglial activation

**DOI:** 10.18632/oncotarget.15325

**Published:** 2017-02-14

**Authors:** Li Li, Hexige Saiyin, Jingmo Xie, Lixiang Ma, Lei Xue, Wei Wang, Weimin Liang, Qiong Yu

**Affiliations:** ^1^ Department of Anesthesiology, Huashan Hospital, Fudan University, Shanghai, 200040, China; ^2^ State Key Laboratory of Genetic Engineering, School of Life Sciences, Fudan University, Shanghai, 200433, China; ^3^ Department of Anatomy, Histology & Embryology, Shanghai Medical College, Fudan University, Shanghai, 200032, China; ^4^ Department of Physiology and Biophysics, School of Life Sciences, Fudan University, Shanghai, 200433, China

**Keywords:** ischemia and reperfusion, microglia, neurogenesis, sevoflurane preconditioning, stem cells

## Abstract

Brain ischemia causes irreversible damage to functional neurons in cases of infarct. Promoting endogenous neurogenesis to replace necrotic neurons is a promising therapeutic strategy for ischemia patients. The neuroprotective role of sevoflurane preconditioning implies that it might also enhance endogenous neurogenesis and functional restoration in the infarct region. By using a transient middle cerebral artery occlusion (tMCAO) model, we discovered that endogenous neurogenesis was enhanced by sevoflurane preconditioning. This enhancement process is characterized by the promotion of neuroblast proliferation within the subventricular zone (SVZ), migration and differentiation into neurons, and the presence of astrocytes and oligodendrocytes at the site of infarct. The newborn neurons in the sevoflurane preconditioning group showed miniature excitatory postsynaptic currents (mEPSCs), increased synaptophysin and PSD95 staining density, indicating normal neuronal function. Furthermore, long-term behavioral improvement was observed in the sevoflurane preconditioning group consistent with endogenous neurogenesis. Further histological analyses showed that sevoflurane preconditioning accelerated microglial activation, including migration, phagocytosis and secretion of brain-derived neurotrophic factor (BDNF). Intraperitoneal injection of minocycline, a microglial inhibitor, suppressed microglial activation and reversed neurogenesis. Our data showed that sevoflurane preconditioning promoted microglial activities, created a favorable microenvironment for endogenous neurogenesis and accelerated functional reconstruction in the infarct region.

## INTRODUCTION

Ischemic stroke is one of the leading causes of morbidity and mortality in elderly people around the world. The devastating consequences result from irreversible neuronal death within the infarct region, leading to significant motor and cognitive disabilities in patients [1]. Restoring neuronal function in the infarct region with endogenous repair and/or replacement by transplantation is considered a promising therapeutic strategy for ischemia and reperfusion injury. In the adult mammalian brain, neural precursor cells residing in SVZ [2] and the subgranular zone (SGZ) [3] proliferate after ischemic insults, migrating into the damaged striatum [4–6] and cortex [6, 7], and ultimately differentiate into neurons, resulting in the limited restoration of the function of the damaged tissues [8]. Although neurogenesis has been observed in the infarct region, newborn neurons only survive for a short period of time due to an unfavorable microenvironment characterized by severely necrotic debris and a lack of neurotrophic support [4]. Therefore, enhancing endogenous neurogenesis and protecting the newborn neurons against the toxic mircoenvironment with therapeutic agents might lead to better recovery in cerebral ischemia.

Based on the reality of the limited self-repairing ability in the ischemic brain, recently developed therapies have focused on either protecting injured neurons or decreasing neuronal apoptosis within the peri-infarct region [9]. For example, pharmacological preconditioning methods using volatile anesthetics can exert direct neuroprotective effects in stroke models [10–12]. In particular, sevoflurane (sevo), a volatile anesthetic that is widely used in clinical surgeries, has been shown to protect damaged neurons by attenuating neuronal apoptosis and inflammation in the peri-infarct region [10, 13]. Sevoflurane is also reported to significantly improve neurological deficits and reduce infarct volumes [14–16]. These findings imply that sevoflurane has the potential to enhance endogenous neurogenesis and the functional reconstruction of the infarct region by creating a favorable microenvironment.

Microglia are critical scavengers and first-line surveillance agents in the central nervous system, with dual roles of neurotoxicity and neuroprotection after ischemic insults. They can rapidly secrete pro-inflammatory factors to trigger inflammation. Meanwhile, microglia phagocytize necrotic cellular debris and secrete neurotrophic factors to protect ischemic neurons [17, 18]. Sevoflurane preconditioning can protect the neurons in the peri-infarct region from apoptosis by reducing inflammation and can also allow for the creation of a favorable microenvironment for endogenous neurogenesis by microglial activities. Thus, we hypothesize that the neuroprotective roles of microglia under sevoflurane preconditioning might prevail over the neurotoxic effects in cases of ischemia and reperfusion injury. It is also possible that sevoflurane might promote microglial activation to phagocytize toxic necrotic debris and secrete neurotrophic factors to create a favorable microenvironment for brain repair.

In this study, we tested our hypothesis that sevoflurane preconditioning enhances endogenous neurogenesis and reconstruction within the infarct zone after ischemic injury. Here, we show that sevoflurane preconditioning promotes the proliferation, migration and differentiation of neuroblasts and that the subsequent neurogenesis is the result of the promotion of microglial neuroprotective activation. Activated microglia phagocytize necrotic debris and secrete BDNF in the sevoflurane-preconditioned brain, consequently creating a favorable microenvironment for functional repairs at the site of infarct after brain ischemia.

## RESULTS

### Sevoflurane preconditioning promoted neuroblasts’ proliferation, migration and differentiation after IR

**Figure 1:**
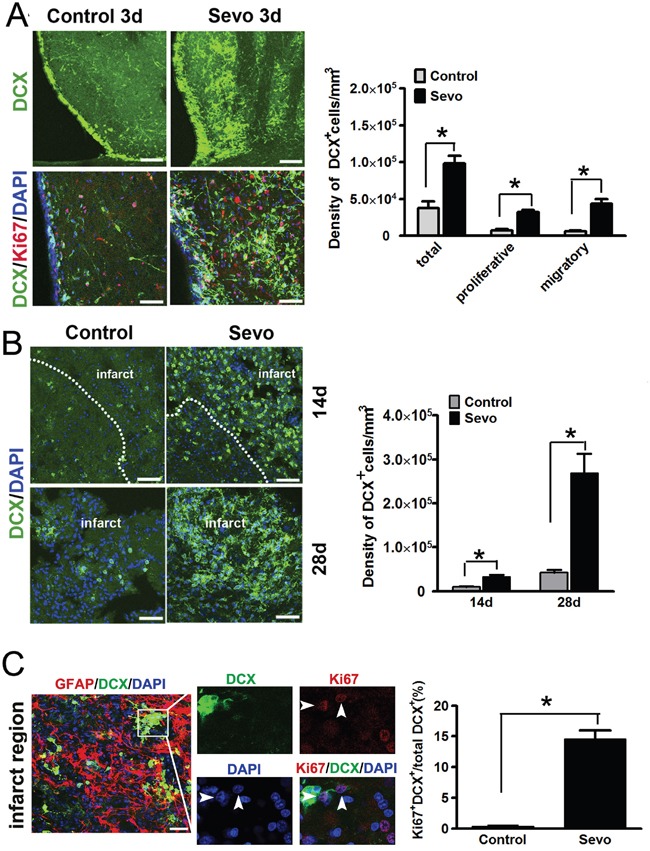
Sevoflurane preconditioning expanded the pool of DCX^+^ cells in the SVZ and accelerated their migration into the infarct **A**. Representative images of DCX^+^ cells in the ipsilateral SVZ in the Sevo and Control groups on day 3, Scale bar, 40μm; Ki67 and DCX immunostaining in the SVZ showed proliferative neuroblasts in both groups on day 3, Scale bar, 20μm; Histogram showed the comparison of different types of DCX^+^ neuroblasts in the SVZ between the two groups. **B**. Representative images of DCX staining in the infarct in the Sevo and Control groups on days 14 and 28. Scale bar, 20μm. Histogram represented quantification of density of DCX^+^ neuroblasts in the infarct of the Sevo and Control groups on days 14 and 28. **C**. Representative images of Ki67 and DCX staining in the infarct region in the Sevo group on day 14 (boxed area indicates clone-like structure; arrow, proliferative cells). Scale bar, 20μm; Histogram showed comparison of the ratio of Ki67-positive neuroblasts to total neuroblasts in the infarct region in Sevo and Control groups.*P<0.05, n=8.

To test if sevoflurane preconditioning has the ability to promote endogenous neurogenesis in the SVZ after ischemic injury, we compared neuroblasts’ proliferation in the SVZ of the Sevo group brains with the Control group brains. Doublecortin (DCX) was used to label the neuroblasts and Ki67 was used for the proliferative cells. Consistent with the observations from other studies [4, 5], we found that the number of DCX^+^ neuroblasts increased in the ipsilateral SVZ in both Control and Sevo groups (Figure 1). Surprisingly, the total number of DCX^+^ cells in the ipsilateral SVZ of the Sevo group was 2.6 times higher than that of the Control group (9.81±1.03×10^4^/mm^3^ vs 3.74±0.91×10^4^/mm^3^, *P*<0.01, n=8) on day 3 after IR (Figure 1A). In particular, a large percentage of the DCX^+^ cells presented the typical morphology of migratory neuroblasts characterized by long, bipolar or unipolar processes in the Sevo group (Figure 1A). The number of migratory neurons in the Sevo group were higher than in the Control group (40.5±4.8% vs 8.1±2.2%, *P*<0.01, n=8). Ki67 and DCX co-staining further showed that 33±6% of DCX^+^ cells were proliferative in the Sevo group, but only 20±2.7% of neuroblasts were both Ki67 and DCX positive in the Control group. These results indicated that sevoflurane preconditioning promoted neuroblasts’ migration and proliferation in the SVZ in the early stages after IR and that these neuroblasts might efficiently migrate to the site of infarct.

To further confirm whether this large number of SVZ-derived neuroblasts could migrate to the infarct region, we evaluated the DCX stained-ischemic brains on days 14 and 28. Consistent with our hypothesis, many DCX^+^ neuroblasts were localized to the infarct regions in the Sevo group while fewer neuroblasts appeared in the Control group (Figure 1B). The number of DCX^+^ neuroblasts in the Sevo group was nearly three times and six times higher than that in the Control group (3.31±0.39×104/mm^3^ vs 0.97±0.09×104/mm^3^; 26.9±4.4×104/mm^3^ vs 4.6±0.6×104/mm^3^, *P*<0.01, n=8) on day 14 and day 28, respectively. Strikingly, even on day 14, we observed that DCX^+^ neuroblasts in the inner side of the infarct regions formed a clone-like structure in the Sevo group and that a significant percentage of these DCX^+^ cells were Ki67 positive, but scarcely any Ki67-positive DCX^+^ cells could be found in the infarct sites of the Control group at the same time (Figure 1C). These results implied that sevoflurane preconditioning not only promoted neuroblast survival, but also accelerated neuroblasts’ migration into the infarct region and their subsequent proliferation.

**Figure 2:**
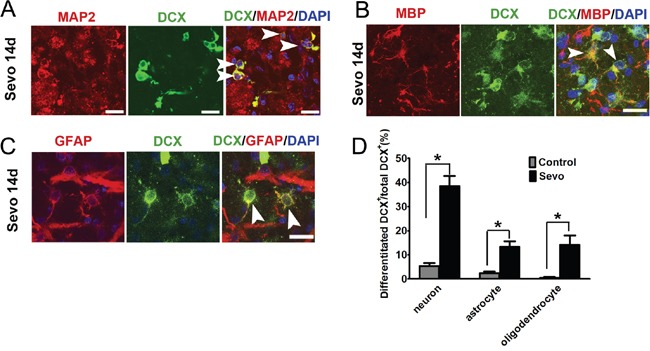
Sevoflurane preconditioning accelerated neuroblasts differentiated into neurons, astrocytes, and oligodendrocytes in the infarct **A**. Image showing DCX^+^ cells that have differentiated into MAP2-positive mature neurons in the infarct in the Sevo group on day 14. Scale bar, 10μm. **B**. DCX^+^ cells differentiated into MBP-positive oligodendrocytes in the infarct in the Sevo group on day 14. Scale bar, 10μm. **C**. DCX^+^ cells differentiated into GFAP-positive astrocytes in the Sevo group on day 14 (white arrow indicates newborn astrocytes). Scale bar, 10μm. **D**. Comparison of differentiation ratio of DCX^+^ cells into neurons or glial cells in the infarct in the Sevo and Control groups; *P<0.05, n=8.

Neurogenesis in the adult brain involves the proliferation and migration of neuroblasts toward target areas and terminal differentiation into distinct brain cell phenotypes [24–26]. Neuroblasts’ differentiation was identified by co-labeling DCX with microtubule associated protein 2 (MAP2) for neurons, glial fibrillary acidic protein (GFAP) for astrocytes or myelin basic protein (MBP) for oligodendrocytes in the infarct regions on day 14, marking neurons, astrocytes and oligodendrocytes. Our results showed that 38.5% of DCX^+^ neuroblasts were MAP2^+^ in the Sevo group (Figure 2A, 2D). Both MAP2^+^ and DCX^+^ cells had typical morphologies with appropriate neuronal polarity. However, only 5.3% of DCX^+^ neuroblasts had differentiated into MAP2^+^-mature neurons in the Control group. Furthermore, we observed that 14% of DCX^+^ neuroblasts were co-labeled with MBP^+^ oligodendrocytes (Figure 2B, 2D) and 13.4% of DCX^+^ cells were co-labeled with GFAP^+^ astrocytes (Figure 2C, 2D). Although neuroblasts differentiated into glial cells in the Control group as well, they accounted for a much smaller percentage (2.4% of DCX^+^ cells with GFAP^+^ and 0.5% of DCX^+^ cells with MBP^+^) when compared to the Sevo group (Figure 2D). These results confirmed that sevoflurane preconditioning promoted neuroblasts’ survival and differentiation after brain ischemic injury.

### Sevoflurane preconditioning promoted axonal regeneration and synapse formation in the infarct after IR

**Figure 3:**
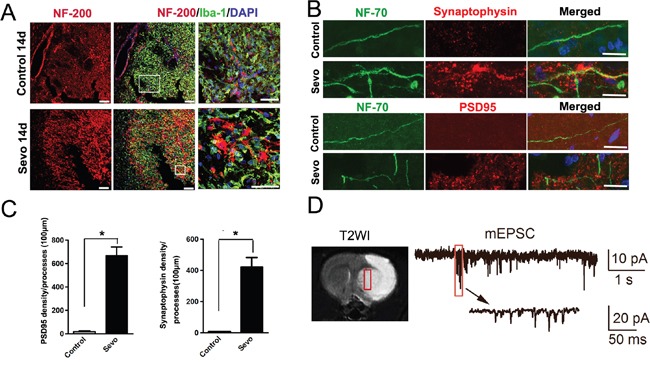
Sevoflurane preconditioning enhanced structural and functional recovery of the ischemic brain **A**. Representative images of the infarct in the Sevo and Control groups that were stained with NF-200 and Iba-1 on day 14. Boxed area indicates magnified region in right panel. Scale bar, 50μm. **B**. High-resolution images of PSD95 or synaptophysin and NF-70 staining in the infarct in the Sevo and Control groups on day 14. Scale bar, 10μm. **C**. Comparison of PSD95 and synaptophysin axonal density in the infarct between the two groups. *P<0.05, n=8. **D**. T2WI image shows the coronal view of brain on day 1, the white tissue showed infarct and the grey tissue showed the healthy tissue on T2WI image. The red outlined region indicates the fixed position for Whole-cell patch clamping of cells; Whole-cell patch clamping of newborn neurons on day 28, showing action potentials and spontaneous inward synaptic currents.

To test whether sevoflurane preconditioning also accelerates axonal growth, we labeled neural axons with neurofilaments 200 (NF-200) on day 14 after IR. The density of NF-200^+^ axons was significantly higher in the inner side of the infarct regions in the Sevo group than in the Control group (Figure 3A). To determine the synaptogenesis induced by sevoflurane preconditioning, we compared and calculated the density of synaptophysin and PSD95, accepted markers for excitatory postsynaptic density. We found abundant synatophysin and PSD95 puncta on NF-70 labeled neurofliaments in the infarct regions in the Sevo group, but the puncta were completely absent in the Control group on day 14 (Figure 3B, 3C). The higher density in the Sevo group indicated that newborn neurons could receive synaptic inputs. To test whether the newborn neurons have action potentials, we confirmed a fixed position via MRI scanning on day 1 after surgery (Figure 3D). Notably, we observed spontaneous mEPSCs in the newborn neurons in the margins of the infarct regions on day 28 (Figure 3D), indicating that the newborn neurons have the characteristics of functional neurons in the infarct regions in the Sevo group. Taken together, these results indicated that sevoflurane preconditioning promoted axonal regeneration and synapse formation in the infarct.

### Sevoflurane preconditioning enhanced microglial migration, phagocytosis and secretion of BDNF after IR

**Figure 4:**
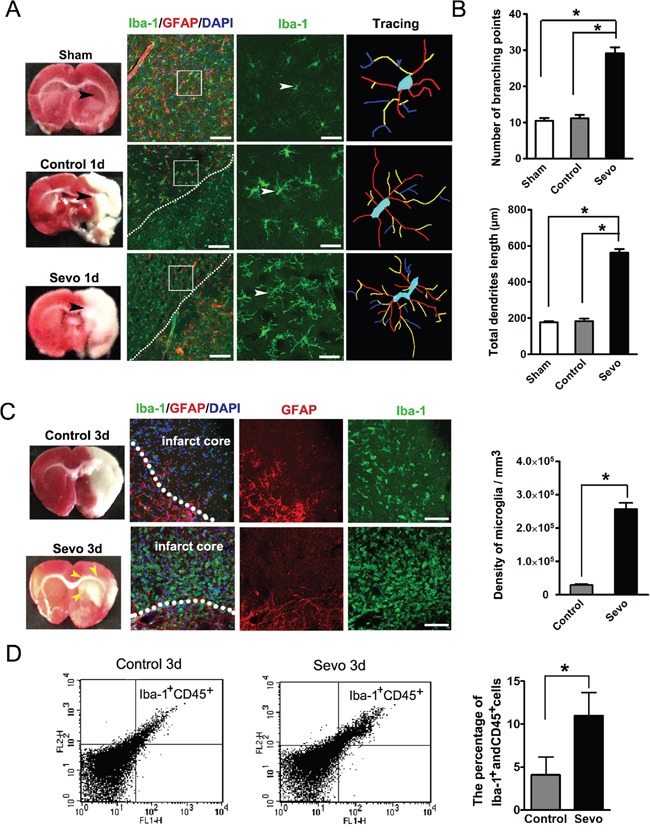
Sevoflurane preconditioning accelerated microglia activated **A**. Comparison of the morphology of Iba-1 stained microglia in the Sevo group with those in Sham and Control groups on day 1. Boxed area indicates magnified region of the middle panels. Scale bar, 40 μm and 10 μm. **B**. Comparison of branching and total dendritic length of microglia across Sham, Control and Sevo groups; *P<0.05, n=8. **C**. Iba-1 stained microglia density in the infarct region both in the Sevo and Control groups on day 3. Dotted lines delineate the boundary of the infarct and the peri-infarct. Yellow arrows indicate the blurry region. Scale bar, 20μm. Histgram showed quantification of microglia density in the infarct on day 3. *P<0.05, n=8. **D**. Brain cells analyzed by flow cytometry displayed patterns of Iba-1 and CD45 expression between the Sevo-and Control-group, The percentages of Iba-1^+^ and CD45^+^ microglia over total cells were quantified and were significantly different; *P<0.05, n=8.

**Figure 5:**
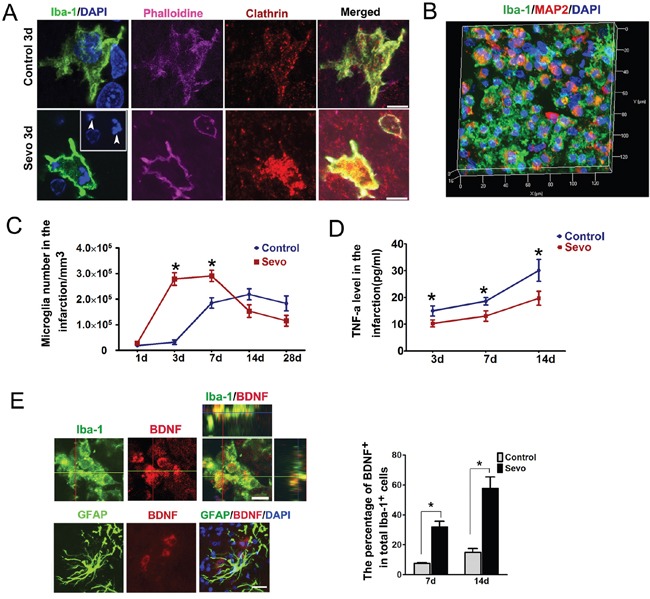
Sevoflurane preconditioning strengthened microglial phagocytosis and secretion of BDNF activities **A**. Phalloidin, Clathrin and Iba-1 stained microglia in the infarct in the Sevo and the Control groups on day 3 (Arrows indicate nuclear debris), Scale bar, 5μm. **B**. Three-dimensional image of inclusion of Iba-1 stained microglia and MAP2 debris in the infarction region in Sevo group on day 3. Histogram represented comparison of activated microglia that phagocytized cell debris between the two groups in the infarct on day 3; ^#^*P<0.05, n=8. **C**. Comparison of ameboid microglia counts in the infarct between the two groups on days 1, 3, 7, 14 and 28; *P<0.05, n=8. **D**. Comparison of TNF-a expression determined by ELISA in the infarct between the two groups on days 3,7 and 14 ; *P<0.05, n=8. **E**. Ortho image demonstrating BDNF secretion by microglia in the infarct in Sevo group, Scale bar, 10μm; Histogram showed comparsion of BDNF-positive microglia in the infarct region in the Sevo group and the Control group on days 7 and day 14; *P<0.05; Representative image showed BDNF was not secreted by GFAP positive astrocytes in the infarct in the Sevo group. Scale bar, 40μm.

Consistent with previous works [10–12], our TTC staining data showed a significant reduction of infarct volumes in the Sevo group compared to the Control group on day 3 after ischemia. Since TTC dye reacts with the mitochondrial enzymes of living cells [27] and microglia migrate to ischemic lesions as first-line surveillance cells [17], we hypothesized that the reduction of infarct volumes might be due to the early migration of activated microglia induced by sevoflurane preconditioning. In this study, we observed that microglia, labeled with Iba-1, changed to a migratory morphology with long processes and more branching points in the peri-infarct zones of the Sevo group on day 1 (Figure 4A, 4B). To demonstrate if over-ramified migratory microglia migrated to the site of infarct, we counted the number of microglia in thick sections after Z-stack scanning with confocal microscopy and reconstructing the 3D structure. Our results showed a large amount of Iba-1^+^ microglia in the infarct regions of preconditioned brains on day 3. Conversely, we only observed a small number of microglia in the untreated brains. We also found that microglia in the infarct regions transformed into the activated ameboid phenotype. The total number of ameboid microglia in the Sevo group was nearly nine times higher than in the Control group (2.57±0.18×10^5^/mm^3^ vs 0.29±0.03×10^5^/mm^3^, *P*<0.01, n=8) (Figure 4C). The expression of CD45 on resting microglia is low, but it is upregulated when microglia become activated [28]. To further characterize the microglial activation levels at the site of infarct, we collected infarct tissue samples at day 3 over Percoll gradients and performed flow cytometry with CD45 and Iba-1 antibodies to assess the activation level. The flow cytometry data showed that the percentage of Iba-1^+^ and CD45^+^ cells in the Sevo group were higher than in the Control group (10.09±2/71% vs 4.09±2.99%, *P*<0.01, n=8) (Figure 4D), which was consistent with our immunostaining results. Microglia phagocytic or endocytic activities rely on strong movements of the actin cytoskeleton in a clathrin- [29] or caveolin-dependent manner [30]. Dysregulation of endocytic activity in the brain has been reported to enhance the brain injury [31]. To directly test whether the microglial phagocytic activities increased, we stained microglia with Iba-1, phalloidin, and clathrin (a marker for specific phagocytic or endocytic activity). The data showed that many clathrin^+^ vesicles within the microglial cytoplasm co-localized with the actin-rich cytoskeleton in the infarct regions of the Sevo group, whereas clathrin was mostly localized in the microglial membrane in the Control group. In addition, we observed that microglia with filopodia-like protrusions engulfed nuclear debris (Figure 5A). To further observe if the activated microglia engulfed dying neurons, we double-labeled mature neurons with MAP2 and Iba-1 to assess microglial phagocytic activities. We observed that most of the ameboid microglia engulfed MAP2^+^ fragments. Sevoflurane significantly increased the microglial engulfment of the cellular debris (Figure 5B). In the Sevo group, the number of activated microglia within the infarct zones increased sharply on day 3, peaked on day 7 after treatment, and began to decline through day 14. In contrast, in the Control group, the number of activated microglia showed a later increase from day 3 to day 7, peaked on day 14, and decreased on day 28 after ischemia (Figure 5C). To detect whether microglial phagocytosis, strengthened by sevoflurane preconditioning, creates a less toxic microenvironment, we evaluated TNF-a, which is the common toxic cytokine from cellular debris of necrotic tissue, with ELISA. Consistent with the observation of more microglia in the infarct regions of the Sevo group, the TNF-a level in the infarction sites of the Sevo group was significantly lower than in the Control group on days 3, 7 and 14 (Figure 5D). The data demonstrated that sevoflurane preconditioning could strengthen microglial migratory and phagocytic activities after ischemia, leading to a favorable microenvironment for neuroblast survival.

Microglia have also been reported to secrete neurotrophins such as BDNF to accelerate neurogenesis and synaptogenesis [32]. In addition, infusion of BDNF into the lateral ventricle of adult rats leads to neurogenesis in the striatum [33]. Thus, to determine if these earlier-arrived microglia secreted BDNF, we triple-stained the infarct regions with BDNF, Iba-1 and GFAP. In Control and Sevo groups, we observed that a significant percentage of microglia, but not astrocytes, were positive for BDNF. Furthermore, the percentage of BDNF-positive microglia in the Sevo group was higher than in the Control group on day 7 and day 14 after IR (Figure 5E).

### Inhibition of microglia activation by minocycline suppressed neuroblasts’ proliferation and migration induced by sevoflurane preconditioning

**Figure 6:**
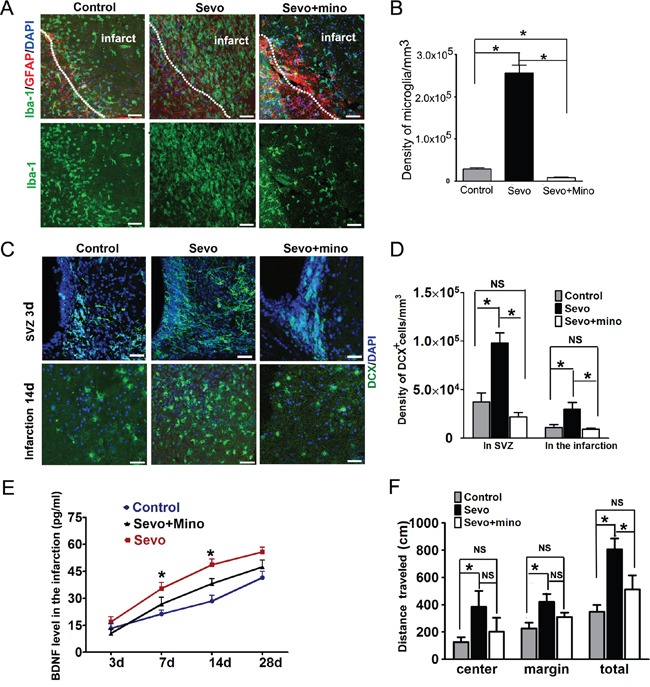
Inhibition of microglia activity by minocycline decreased DCX^+^ cells proliferation and migration **A**. Iba-1 immunostained microglia in the Control, Sevo and Sevo+mino-treated groups in the infarct on day 3. **B**. Comparing the number of microglia in the infarct of Sevo+mino-treated with that in the Sevo and Control groups. Scale bar, 20μm. One-way ANOVA, post-hoc, *P<0.05, n=8. **C**. DCX^+^ cells in the SVZ of Control, Sevo and Sevo+mino-treated group. **D**. Comparing the number of DCX^+^ cells of Sevo+mino-treated with that in the Sevo and Control groups, *P<0.05;NS=Non Significant, n=8. **E**. Comparison of BDNF expression determined by ELISA in the infarct of Control, Sevo and Sevo+mino-treated groups on days 3, 7, 14 and 28; *presentive Sevo vs Control and Sevo vs Sevo+mino, *P*<0.05, n=8. **F**. In the open-field task,. Comparison of the distance traveled of Control, Sevo and Sevo+mino-treated groups on days 28; *P<0.05; NS=Non Significant; n=8.

To further determine whether an increase in neuroblasts' proliferation and migration could be directly due to microglial activation, we administered minocycline, a selective microglial antagonist that does not directly affect the activities of neuroblasts or neurological behavior [34], 30 minutes before MCAO surgery to sevoflurane-preconditioned rats. On day 3, a limited number of microglia appeared within the infarct regions. The number of microglia in the minocycline-treated group decreased by 96.8% (*vs*. Sevo group, *P*<0.001, n=8) and 72.0% (*vs*. Control group, *P*<0.001, n=8) (Figure 6A, 6B). We further analyzed the neuroblasts in minocycline-treated brains and found that the total number of DCX^+^ cells in the SVZ in the minocycline-treated group were 78.2% (vs Sevo group, n=8) and 42.8% (vs Control group, n=8) lower on day 3. Similarly, we observed a smaller number of DCX^+^ neuroblasts in the infarct regions of the minocycline-treated group on day 14 when compared with the Control and Sevo groups, respectively (Figure 6C, 6D). To test whether minocycline administration also decreased the BDNF level, we detected the level of BDNF in the infarct regions by ELISA. Consistent with immunostaining results, the BDNF level in the Sevo group was higher than in the Control group. It was also higher than that in the Sevo+minocyline group on days 7 and 14, but the difference of BDNF levels between Control and Sevo+minocyline groups was not significant (Figure 6E). Minocyline alone does not affect the activities of neuroblasts *in vivo* [34] and did not improve neurological behavior in ischemic rats [35]. Our data further suggest that activated microglia in the infarct region contribute to neurotrophic microenvironments for newborn neuron survival.

Based on the observation of more neurogenesis in the Sevo group, we hypothesized that the neurogenesis in the Sevoflurane-preconditioned brain might have a long-term benefit of neurobehavioral recovery. To examine whether neurogenesis and reconstruction in the infarct region could lead to neurobehavioral improvements, spontaneous locomotor activity was tested in the open-field behavioral task. The center distance, margin distance and total amount of distance traveled were recorded in the chamber. On days 7 and 14, there were no significant differences in distance traveled (not shown). However, on day 28, we found that sevoflurane preconditioning significantly increased the distance traveled, including center distance, margin distance and total distance, compared to the Control group. A modest, statistically significant difference in total distance traveled was observed between Sevo and Sevo+mino groups. Similarly, no differences were observed between the Control group and the Sevo+mino group (Figure 6F). These data suggest that the neurogenesis and reconstruction in the infarct region by Sevo preconditioning contribute to better neurobehavioral recovery after ischemia and reperfusion injury in rats.

## DISCUSSION

A pool of neuroblasts in the SVZ of the adult brain is stimulated by cerebral ischemia and neurodegeneration, with some of them demonstrating the ability to migrate and differentiate into mature neurons. However, most newborn neurons are too vulnerable to survive in a post-infarct environment that includes exposure to products of damaged tissues and/or lack of proper neurotrophic factor support [4]. Therefore, how to create optimal microenvironments for neurogenesis against cerebral ischemia is the top priority for functional replacement of dead neurons with endogenous ones or transplanted ones. In this work, we provide an applicable strategy to enhance endogenous neurogenesis and survival of newborn neurons by enhancing the neuroprotective activity of microglia via sevoflurane preconditioning.

Sevoflurane, a widely used inhalational anesthetic in clinics, has been shown to improve neurological deficits and reduce infarct volumes in experimental models of ischemic stroke in rodents [14–16]. In this study, we showed that repeated and intermittent administration of 1.2% sevoflurane for four consecutive days prior to an ischemic insult not only prolonged overall survival but also improved neurologic recovery for 28 days. Consistent with better neurological recovery in the Sevo group, we observed more neuroblast proliferation in the SVZ and migration into the infarct regions in the Sevo group than in the Control group. More neuroblasts were differentiated into neurons, astrocytes and oligodendrocytes in the infarct sites of the Sevo group than in the Control group. Co-staining of NF-70/PSD95, NF-70/synaptophysin, and mEPSCs showed that newborn neurons are functional. These data indicated superior neurological recovery on day 28 in the Sevo group resulting from endogenous neurogenesis.

The biggest challenge for ischemic stroke patients is not only the initial loss of neuronal function but also the secondary damage to peri-infarct tissues resulting from large amounts of toxic cellular debris as well [8]. Several studies have tried transplanting stem cells, including neural stem/precursor cells [36], embryonic stem cells [37] and human fetal neural stem cells [38], to replace necrotic neurons and repair the infarct region. They showed that the survival, migration and differentiation of the transplanted stem cells are dependent on the microenvironment in the central nervous system. Although the role of microglia in ischemic injury or neurodegeneration is still controversial, their scavenging and secretion of neurotrophic factors are well-defined in the central nervous system [39, 40]. Liposaccharide (LPS)-activated microglia, the classically activated microglia, impair neurogenesis, but the microglia activated by cytokines associated with adaptive immunity are neuroprotective [41]. Microglial cells are activated after ischemic insults and exert neuroprotection by removing toxic debris in the infarct region [42, 43]. Consistent with increased neuroblast activation and proliferation in the Sevo group, we observed more activated microglia migrating towards and infiltrating into the infarct regions in Sevoflurane preconditioned rats on day 3 than in the Control group. Iba-1 and MAP2 co-staining and flow cytometry of Iba-1 and CD45 antibodies demonstrated that the early accumulation of more phagocytic microglia allowed for engulfment of toxic, necrotic neural debris within the infarct region and decreased the level of TNF-a, which could create an optimal microenvironment for neuroblasts' proliferation, migration and survival. BDNF contributes to the promotion of angiogenesis, brain plasticity and enhanced functional recovery after stroke [44]. Intravenous administration of BDNF during the 5 days following cortical stroke is associated with enhanced migration of progenitor cells from the SVZ [45]. We found that Sevoflurane preconditioning also promotes microglial secretion of BDNF on day 7 after ischemia, providing neurotrophic factor support for subsequent neurogenesis. Minocycline is a selective microglial inhibitor that does not affect the neuroblasts' proliferation or differentiation *in vivo* and shows no therapeutic effects in ischemic injury [34]. This agent reversed sevoflurane-induced endogenous neurogenesis. These findings indicate that sevoflurane preconditioning enhances endogenous neurogenesis *via* promotion of both phagocytic activity and secretion of neurotrophic factors from microglia. In clinical practice, sevoflurane preconditioning has been applied before coronary arterial surgery with evidence of cardioprotection [46]. Based on our findings, the illustration of the molecular mechanism by which sevoflurane preconditioning activates the neuroprotective activity of microglia will provide more selectable strategies for better outcomes in ischemic patients in the future.

Thus, sevoflurane preconditioning enhanced brain endogenous reparation after IR injury, partly by activating microglial migration, phagocytosis and secretion of neurotrophic factors. If sevoflurane preconditioning demonstrates enhanced endogenous neurogenesis in primate models in the future, it will provide a new strategy to improve the outcomes in patients with ischemic brain disease.

## MATERIALS AND METHODS

### Experimental groups and sevoflurane preconditioning

Male Sprague-Dawley rats (280–320g, SLAC Experimental Animal Co. Ltd, Shanghai, China) were randomly according to computer-generated random numbers allocated into three groups: Sham, Control and sevoflurane preconditioning (Sevo) groups. In Sevo group, rats were exposed for 60 min on 4 consecutive days to 1.2% sevoflurane (Baxter)+98% O_2_ in an anesthetic chamber. The Control group rats were exposed to 98% O_2_ instead. After 24 hours of treatment, the rats of Control and Sevo groups were subjected to tMCAO. Sham group rats were neither exposed to sevoflurane, nor induced ischemia. All procedures were in accordance with the Guide for the Care and Use of Laboratory Animals and approved by the Committee of Animal Research, Fudan University and followed the ARRIVE guidelines.

### Transient middle cerebral artery occlusion

Transient focal cerebral ischemia was induced by right MCAO as previously described [19]. Briefly, rats were anesthetized with 40 mg/kg ketamine (i.p. injection) and allowed to breathe spontaneously. After the right common carotid artery was ligated, a 0.38±0.02 mm diameter monofilament (Sunbio Biotech Co. Ltd., Beijing, China) was inserted into the right common carotid artery and advanced along the internal carotid artery until occluding the origin of the MCA. After 90 min, the monofilament was withdrawn to establish reperfusion and the wound sutured. Rectal temperature was maintained at 37±0.2 °C with a heating pad perioperatively. After recovery from anesthesia, rats were placed back into cages with free access to food and water.

### Magnetic resonance imaging paremeters

MRI with 3.0 Tesla superconducting magnet(GE MEDICAL SYSTEMS, DISCOVERY MR750) was conducted in the protocol. The rats were fastened to 4-channel phased array coil after anesthetized with 2% pentobarbital sodium. The scanning parameters were as follows: T2W1(TR, 4762 ms; TE, 98.7 ms; flip angle, 111; NEX, 2; slice thickness, 1.8 mm; slice gap, 2 mm;matrix, 512*512), DWI(b 0, 800s/mm2; TR, 2000 ms; TE, 77.8 ms; flip angle, 90; NEX, 8; slice thickness, 1.8 mm; slice gap, 2 mm, matrix, 256*256). The MapIt sequence was used with eight echoes for T2 mapping. The parameters were as follows TE12.9, 25.7, 38.6, 51.4, 64.3, 77.1, 90, 102.8 ms, TR1500 ms, flip angle, 90; NEX, 1; slice thickness, 1.8 mm; slice gap, 2 mm, matrix, 512×512.

### Open field test

The open-field test was used to determine general activity levels. Animals were monitored under moderate lighting for 20 min in a 90-cm^2^ open field using videotracking software (ANY-Maze, Stoelting, IL, USA). General activity was evaluated by determining margin distance, center distance and the total of distance traveled.

### Immunofluorescence analysis

After removing and slicing rat brains, brain slices were stained with 1%TTC, fixed with 4% PFA, and subsequently dehydrated with 30% sucrose in 0.01 M PBS for 24 h at 4°C. The TTC stained slices were embedded in OCT compound for 30min at −20°C and sectioned with 45μm thickness (coronal sections) by cryostat (Leica CM 900, Leica, Germany). Floating brain sections were preserved in the cryoprotectant buffer (30% sucrose + 30% glycol prepared in PBS buffer). The slides were incubated with primary antibodies (Table 1) in dilute solution (0.2% Triton X-100 and 5% goat serum in 0.01 M PBS) overnight at 4°C, and then incubated with fluorescent-labeled secondary antibodies (1:1000, Jackson, West Grove, PA). Nuclei were counter stained by DAPI. All images were scanned with Leica SP8 (Leica Microsystems) or Zeiss LSM 710 (Carl Zeiss) confocal microscopes with Z-stacks, and Z-stacked with Zen 2009 or image J software. Cellular morphology was analyzed by Image J which plugged with simple neurite tracer [20]. The neuroblasts with elongated chain-like process was defined as migratory neuroblasts [4, 5]. At each time point, we have 8 rats in per group.

**Table 1:** Antibodies list

Antibody	Isotype	Dilution	Source
Iba-1	Goat IgG	1:1000	Abcam
GFAP	Mouse IgG	1:1000	Abcam
GFAP	Rabbit IgG	1:5000	DAKO
MBP	Rabbit lgG	1:500	Abcam
MAP2	Rabbit lgG	1:1000	Sigma
DCX	Goat lgG	1:250	Santa cruz
NF-70	Mouse lgG	1:1000	Millipore
NF-200	Mouse IgG	1:500	Abcam
Ki67	Rabbit lgG	1:100	ZYMED
PSD95	Rabbit lgG	1:1000	Abcam
Synaptophysin	Rabbit lgG	1:1000	Abcam
Clathrin	Mouse IgG	1:500	Abcam
BDNF	Rabbit lgG	1:200	Boster
Phalloidine		1:10000	Sigma
DAPI		1:10000	Abcam

### Enzyme-linked immunosorbent assay (ELISA) Analysis

The infarction region of brains was isolated from the whole brain slices after TTC staining as previous described [21]. The total protein in each sample was determined using the bicinchoninc acid method (BCA; Pierce Biotechnology, Inc.).

### Isolation of single cells and fluorescence-activated cell sorting(FACS)

After sacrificing the rats, the infarct region was isolated from the whole brain and transferred to ice-cold Hanks' buffer, cut by stainless steel scissors to small pieces and each brain was digested by 0.25% Trypsin-EDTA for 20 min at 37°C. Isolated cells were incubated on ice for 30 min with anti-goat Iba-1 and CD45-Alex488. After washes, cells were then incubated on ice for 40min with fluorescent-labeled PE secondary antibodies (1:1000, Jackson, West Grove, PA). After washes, fixed in 1% paraformaldehyde. FACS was performed with a FACS Calibur flow cytometer and analyzed using Cell Quest Pro (BD Biosciences, San Diego, CA, USA)

### Whole-cell patch-clamp recording

The neurons were collected in a whole-cell patch configuration as previous described [22, 23]. Briefly, the rat striatal brain slices according to fixed position by MRI scanning at 24h after surgery were prepared using a vibrotome (Leica VT 1200S, Germany). The slices were incubated in artificial cerebrospinal fluid for 30 min at 37°C and then held at room temperature (22-24°C) for the duration of the recordings. The bath solution contained (in mM): 125 NaCl, 2.5 KCl, 25 NaHCO_3_, 3 myo-inostol, 2 Na-pyruvate, 1.25 NaH_2_PO_4_, 0.4 ascorbic acid, 25 D-glucose, 1 MgCl_2_, 2 CaCl_2_, 0.001 TTX, pH 7.4 when bubbled with 95% O_2_ and 5% CO_2_. Postsynaptic pipette (2 - 3 MΩ) solution contained (in mM): 125 K-gluconate, 20 KCl, 4 MgATP, 10 Na_2_-phosphocreatine, 0.3 GTP, 10 HEPES, and 0.5 EGTA, pH 7.2, adjusted with KOH (osmolarity was 310 - 320 Osm). To isolate AMPA receptor-mediated mEPSCs, blockers of NMDA receptors (D-APV, 50 μM), GABA_A_ receptors (bicuculline, 10 μM) and glycine receptors (strychnine, 10 μM) were added to the bath solution. The immature granule cells usually have larger input resistance, which exceeds gigaohm. Most cells we recorded in the infarct ranged from 0.9 GΩ ~ 1.2 GΩ with their input resistences [23].

### Minocycline treatment

30 minutes before MCAO surgery, minocycline (45 mg/kg; i.p., Sigma–Aldrich, St. Louis, MO, USA) was administrated to the preconditioned rats, and sacrificed on days 3 and day 14 (n=8 per group). Rats of Control group were given saline instead.

### Statistical analysis

Statistical analysis was performed in Graphpad Prism 5 or SPSS 20. The difference between two groups was analyzed with Student's *t* test. For multigroup comparisons, one-way ANOVA with post-hoc was used. Overall survival was estimated by Kaplan merrier method. All data are presented as mean ± SD unless otherwise specified. All data collection were performed with double blind way. *P* values less than 0.05 were considered statistically significant. “n” refers to the number of rats.

## Abbreviations

IR: ischemia and reperfusion
tMCAO: transient Middle Cerebral Artery Occlusion
SVZ: Subventricular Zone
Sevo: Sevoflurane
ELISA: Enzyme-linked immunosorbent assay
BDNF: brain-derived neurotrophic factor
